# The roles of *MMP8*/*MMP10* polymorphisms in ischemic stroke susceptibility

**DOI:** 10.1002/brb3.2797

**Published:** 2022-10-25

**Authors:** Yong Zhao, Qi Zhang, Xiaobo Zhang, Yu Zhang, Ying Lu, Xiaojuan Ma, Weiping Li, Xiaochen Niu, Gejuan Zhang, Mingze Chang, Wenzhen Shi, Ye Tian

**Affiliations:** ^1^ Department of Neurology, Xi'an Key Laboratory of Cardiovascular and Cerebrovascular Diseases, Xi'an No. 3 Hospital the Affiliated Hospital of Northwest University Xi'an Shaanxi China; ^2^ The College of Life Sciences Northwest University Xi'an Shaanxi China; ^3^ Medical Research Center, Xi'an Key Laboratory of Cardiovascular and Cerebrovascular Diseases, Xi'an No. 3 Hospital the Affiliated Hospital of Northwest University Xi'an Shaanxi China

**Keywords:** genetic polymorphisms, ischemic stroke, MMP8/MMP10, susceptibility

## Abstract

**Background:**

Ischemic stroke (IS), a multifactorial and polygenic disease, is the most common cause of death. This study aimed to determine the roles of *MMP8*/*MMP10* polymorphisms in IS susceptibility in the Chinese Han population.

**Methods:**

*MMP8* rs1940475 and rs3765620, and *MMP10* rs17860949 from 700 IS patients and 700 controls were genotyped by the MassARRAY iPLEX platform. The impact of polymorphisms on IS risk was evaluated by logistic regression analysis.

**Results:**

Our study indicated that rs17860949 in *MMP10* was significantly associated with a reduced risk of IS (OR = 0.632, *p* = .002). Precisely, stratification analysis showed that rs17860949 was relate to a decreased susceptibility to IS in patients aged > 55 years (OR = 0.472, *p* < .001), males (OR = 0.632, *p* = .012), nonsmokers (OR = 0.610, *p* = .017), and nondrinkers (OR = 0.559, *p* = .006). All these significant findings were verified by false‐positive report probability test. Furthermore, GG genotype and AG genotype in *MMP8* rs3765620 polymorphism were related to a reduced triglycerides concentration (*p* = .018).

**Conclusion:**

Our study suggests that rs17860949 in *MMP10* may play a protective role in IS in the Chinese Han population.

## 1 INTRODUCTION

Stroke is the most common severe manifestation of cerebrovascular disease and the second leading cause of death in the world with high disability, mortality, and morbidity (Guzik & Bushnell, 2017; Strong et al., 2007). Stroke is divided into ischemic stroke (IS) and hemorrhagic stroke, and the former accounts for about 87 % of total strokes. According to the Report on Cardiovascular Health and Diseases in China, the incidence of stroke in China was 246.8/100,000 in 2019, with males (266.4/100,000) higher than females (226.9/100,000), and rural areas (298.2/100,000) significantly higher than urban areas (203.6/100,000) (The Writing Committee of the Annual Report on Cardiovascular Health and Diseases in China, 2021). Risk factors for IS include age, gender, smoking, alcohol consumption, hypertension, diabetes, and so on (Au, 2018). Although stroke has been identified as a cerebrovascular obstruction caused by atherosclerosis (Weber & Noels, 2011), the pathogenesis underlying IS was not fully understood. Furthermore, it is accepted that IS is a polygenic and multifactorial disease caused by the combined action of genetic and environmental factors (Wei et al., 2015). Over the past few years, many studies have indicated that genetic polymorphisms exert an important role in IS of atherosclerotic origin (Misra et al., 2018). Numerous genetic susceptibility variants for stroke have been identified in recent studies, such as *ACE* (Goyal et al., 2021), *XPF* (Ma et al., 2016), *ITGA2* (Jalel et al., 2021), *MMP9* (Wang et al., 2018), *MMP2* (Christodoulou et al., 2020), and *ESR1* (Fu et al., 2019). Among these variants, matrix metalloproteinases (MMPs) have a vital role in the occurrence of stroke (Kaplan et al., 2008; Manso et al., 2010; Park et al., 2007).

**FIGURE 1:**
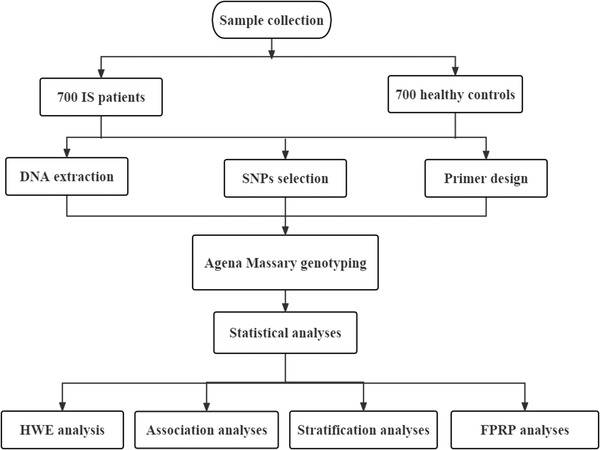
Flow chart of the study

MMPs are a unique family of extracellular calcium‐ and zinc‐binding endopeptidases, which can accelerate atherosclerosis by degrading extracellular matrix in IS patients (Abilleira et al., 2006; Chang et al., 2016). Previous researches have revealed that *MMP* genes play an important role in the pathogenesis of stroke (Ohshima et al., 2010; Schäfers et al., 2010; Su et al., 2005). Moreover, polymorphisms of *MMP* genes, such as *MMP1* (Ghilardi et al., 2002), *MMP2* (Nie et al., 2014), *MMP3* (Ghilardi et al., 2002), *MMP9* (Yuan et al., 2013), and *MMP12* (Wen et al., 2014), are associated with IS risk. Matrix metalloproteinase‐8 (MMP8) is a type of collagenases in the MMPs. Matrix metalloproteinase‐10 (MMP10) is a subgroup of stromelysins in the MMPs. The *MMP8* and *MMP10* genes are also known to be related to the occurrence and development of IS (Lenglet et al., 2013; Navarro‐Oviedo et al., 2019; Purroy et al., 2018). *MMP8* polymorphisms are risk factors for many human diseases, such as breast cancer (Wang et al., 2018), ankylosing spondylitis (Meng et al., 2018), gastric adenocarcinoma (Lin et al., 2017), and osteoarthritis (Näkki et al., 2016). To our best known, the roles of *MMP8* polymorphisms in IS susceptibility are unclear. There have been few studies on the role of *MMP10* polymorphisms in IS. Zhu et al. have found that *MMP10* polymorphisms (rs17435959 and rs17293607) are not associated with the risk of atherothrombotic cerebral infarction in Jiangsu population (Zhu et al., 2013). However, a latest study by Zhou has indicated that *MMP10* polymorphisms are correlated with the susceptibility and formation of carotid atherosclerosis plaques (Wu et al., 2021). In order to obtain more accurate estimation of the polymorphisms of studied genes, we carried out a case‐control study. In our present study, we tried to study the influence of *MMP8*/*MMP10* polymorphisms on IS susceptibility in the Shaanxi Han population. The flow chart of this study IS summarized in Figure 1.

## 2 MATERIALS AND METHODS

### 2.1 Study population

Our study was approved by the ethics committee of Xi'an No. 3 Hospital, the Affiliated Hospital of Northwest University (SYXSLL‐2019‐034), and informed written consent was signed and obtained from all individuals before the study began. From January 2019 to November 2021, we recruited 1400 (700 IS patients and 700 healthy volunteers) unrelated Chinese Han population included from Xi'an No. 3 Hospital, the Affiliated Hospital of Northwest University. Patients were first diagnosed with IS by two experienced neurologists based on clinical symptoms, cerebral scanner, magnetic resonance imaging (MRI), and/or computed tomography according to the guideline for stroke (Liberman et al., 2016). The patients with a history of stroke, genetic diseases, brain tumor or any types of cancers, autoimmune, cardiogenic, and neurological diseases were excluded. Additionally, all healthy controls with no history of cerebrovascular disease were randomly selected and they underwent physical examination at the same period as cases. The characteristics of all participants (age, gender, smoking, alcohol intake, levels of triglycerides, total cholesterol, low‐density lipoprotein cholesterol [LDL‐c], and high‐density lipoprotein cholesterol [HDL‐c]) were obtained from questionnaires or medical records. After obtaining the informed consent of all participants, peripheral blood samples from each individual were harvested at the time of initial diagnosis. A total of 5 ml fasting peripheral venous blood (arm vein) was collected from each patient under aseptic operation in the morning. The blood samples were placed in EDTA‐containing vacutainer tubes and stored at −80°C for DNA extraction and genotyping.

### 2.2 DNA extraction and SNP genotyping

We selected three single nucleotide polymorphisms (SNPs) (rs1940475 and rs3765620 in *MMP8*, and rs17860949 in *MMP10*) from the 1000 Genomes Project database with minor allele frequency (MAF) large than 0.05. Genomic DNA in peripheral venous blood samples was extracted by DNA extraction kit. PCR primers for SNPs genotyping were designed by Agena Bioscience Assay Design software and listed in Table 1. The genotyping of *MMP8*/*MMP10* genetic polymorphisms was identified by the Agena MassARRAY iPLEX platform. Additionally, analysis of the genotyping data was conducted by the Agena Bioscience TYPER 4.0 software.

### 2.3 Bioinformatics analysis

We used HaploReg v4.1 online software (https://pubs.broadinstitute.org/mammals/haploreg/haploreg.php) to predict the possible protein functions in these SNPs.

### 2.4 Statistical analysis

Statistical analysis was processed by SPSS version 20.0, and the significance threshold was set at *p* < .05. Continuous variables such as age, total cholesterol, triglycerides, HDL‐c, and LDL‐c were compared by a Student's *t*‐test. Discrete variables including gender, smoking, alcohol intake, and HWE were tested using the chi‐square test. The impacts of *MMP8*/*MMP10* polymorphisms on IS susceptibility were determined by multiple logistic regression models (allele, codominant, dominant, recessive, and log‐additive) with adjustments for age, gender, smoking, and alcohol intake. We also detected the association of SNPs with risk factors for IS after stratification by age, gender, smoking, and alcohol intake. Besides, a false‐positive report probability (FPRP) analysis was used to verify the positive findings in the current study (Deng et al., 2020). Furthermore, we detected the influence of *MMP8*/*MMP10* genetic variants on the risk factors for IS using one‐way analysis of variance (ANOVA).

## 3 RESULTS

### 3.1 Characteristics of study population

This study involved 700 IS patients (459 males and 241 females) and 700 healthy controls (457 males and 243 females). The mean ages of IS patients and healthy controls were 55.02 ± 6.79 years and 55.74 ± 9.14 years, respectively. As shown in Table 2 , levels of total cholesterol, HDL‐c, and LDL‐c in cases were significantly lower than these in controls (*p* < .001, *p* = .035, and *p* < .001, respectively). In terms of age, gender, smoking status, alcohol intake, and triglycerides, there was no statistically significant difference between the case and control groups (*p* = .095, *p* = .955, *p* = .957, *p* = .707, and *p* = .510, respectively).

**TABLE 1:** Primers used for this study

SNP_ID	1st PCRP	2nd PCRP	UEP DIR	UEP SEQ
rs1940475	ACGTTGGATGTTTGGGTTGAATGTGACGGGGC	ACGTTGGATGTAAAACCACCACTGTCAGGCTC	F	CTCCACAGCGAGGCTTTT
rs3765620	ACGTTGGATGCATCACCTAACTGATAGTTC	ACGTTGGATGCATGTGCAGATTTCCAAGGC	R	TCTTCTAAAGAGAAAAATACAAAAACCA
rs178609499	ACGTTGGATGCTGACGTTGGTCACTTCAGC	ACGTTGGATGCTAATCTGAACCATTACCTGC	F	TCTGAACCATTACCTGTATGTAAGC

SNP, single nucleotide polymorphisms; PCRP, polymerase chain reaction primer; UEP‐DIR, unextension primer sequence direction; UEP SEQ, unextended mini‐sequencing primer sequence.

1st PCRP means the first PCR primer. 2nd PCRP means the second PCR primer.

**TABLE 2:** Basic characteristics of participants in this study

Variables	Cases (*n* = 700)	Controls (*n* = 700)	*p*
Age, years (mean ± SD)^a^	55.02 ± 6.79	55.74 ± 9.14	.095
>55	398 (56.9 %)	309 (44.1 %)	
≤55	302 (43.1 %)	391 (55.9 %)	
Gender^b^			.955
Male	459 (65.6 %)	457 (65.3 %)	
Female	241 (34.4%)	243 (34.7 %)	
Smoking status^b^			.957
Smoker	337 (48.1 %)	339 (48.4 %)	
Nonsmoker	363 (51.9 %)	361 (51.6 %)	
Alcohol intake^b^			.708
Yes	337 (48.1 %)	345 (49.3 %)	
No	363 (51.9 %)	355 (50.7%)	
Total cholesterol (mmol/L)^a^	3.96 ± 1.00	4.81 ± 0.93	<.001
Triglycerides (mmol/L)^a^	1.75 ± 1.11	1.81 ± 1.18	.510
HDL‐c (mmol/L)^a^	1.10 ± 0.24	1.15 ± 0.29	.035
LDL‐c (mmol/L)^a^	1.97 ± 0.68	2.66 ± 0.73	<.001

^a^ Student's *t*‐test is used.

^b^ Pearson′s *X^2^* test is used.

The *p* < .05 indicates statistical significance.

HDL‐C, high‐density lipoprotein cholesterol; LDL‐C, low‐density lipoprotein cholesterol.

### 3.2 Association of *MMP8*/*MMP10* polymorphisms with IS susceptibility

Three SNPs (included rs1940475 and rs3765620 in *MMP8*, and rs17860949 in *MMP10*) were detected in this study. Table 3 presents the details and potential functions of these SNPs. The frequency distributions of rs1940475, rs3765620, and rs17860949 genotypes in controls met the Hardy–Weinberg equilibrium (*p* = .487, *p* = .923, and *p* = .837, respectively). In addition, five genetic models were applied to investigate the influence of the three studied SNPs on IS risk. As exhibited in Table 4, rs17860949 was significantly associated with a decreased risk of IS in the allele (OR = 0.632, 95% CI = 0.469–0.853, *p* = .002), codominant (OR = 0.641, 95% CI = 0.467–0.879, *p* = .006), dominant (OR = 0.627, 95% CI = 0.458–0.858, *p* = .004), and log‐additive models (OR = 0.627, 95% CI = 0.463–0.850, *p* = .003).

**TABLE 3:** The distribution of allele frequencies of MMP8/MMP10 SNPs

				MAF			
SNP ID	Gene	Chromosome position	Alleles (A/B)	Cases	Controls	*p* ^a^‐HWE	HaploReg v4.1
rs1940475	MMP8	chr11: 102722517	T/C	0.103	0.097	0.487	Enhancer histone marks, DNAse, Motifs changed, GRASP QTL hits, Selected eQTL hits
rs3765620	MMP8	chr11: 102724761	G/A	0.228	0.238	0.923	Enhancer histone marks, DNAse, Motifs changed, GRASP QTL hits, Selected eQTL hits
rs17860949	MMP10	chr11: 102779515	A/G	0.229	0.221	0.837	Enhancer histone marks, Motifs changed

SNP, single nucleotide polymorphisms; A, minor allele; B, major allele; MAF, minor allele frequency; HWE, Hardy–Weinberg equilibrium.

The *p* ^a^ < .05 are excluded.

**TABLE 4:** Association of MMP8/MMP10 polymorphisms with ischemic stroke risk

SNP ID	Model	Allele/genotype	Case N	Control N	OR (95% CI)	*p*
rs1940475	Allele	*C*	873	881	1	
		*T*	521	557	1.017 (0.872–1.186)	.830
	Codominant	*TC*	311	323	0.955 (0.760–1.199)	.690
		*TT*	105	97	1.079 (0.782–1.790)	.644
		*CC*	281	279	1	
	Dominant	*TC*‐*TT*	416	420	0.983 (0.794–1.218)	.878
	Recessive	*CC*‐*TC*	592	602	1	
		*TT*	105	97	1.106 (0.820–1.491)	.509
	Log‐additive	–	–	–	1.018 (0.875–1.184)	.821
rs3765620	Allele	*A*	891	897	1	
		*G*	507	503	1.015 (0.870–1.184)	.853
	Codominant	*AG*	299	315	0.936 (0.746–1.174)	.565
		*GG*	104	94	1.092 (0.791–1.508)	.594
		*AA*	296	291	1	
	Dominant	*AG*‐*GG*	403	409	0.972 (0.785–1.202)	.790
	Recessive	*AA*‐*AG*	595	606	1	
		*GG*	104	94	1.130 (0.836–1.527)	.428
	Log‐additive	–	–	–	1.016 (0.874–1.182)	.835
rs17860949	Allele	*G*	1318	1283	1	
		*A*	76	117	0.632 (0.469–0.853)	**.002**
	Codominant	*AG*	74	109	0.641 (0.467–0.879)	**.006**
		*AA*	1	4	0.246 (0.027–2.212)	.211
		*GG*	622	587	1	
	Dominant	*AG*‐*AA*	75	113	0.627 (0.458–0.858)	**.004**
	Recessive	*GG*‐*AG*	696	696	1	
		*AA*	1	4	0.261 (0.029–2.349)	.231
	Log‐additive	–	–	–	0.627 (0.463–0.850)	**.003**

CI, confidence interval; OR, odds ratio; SNP: single nucleotide polymorphism; OR, odds ratio, 95 % CI; 95% confidence intervals.

The *p* values were calculated by unconditional logistic regression analysis with adjustments for age, gender, smoking, and drinking.

The *p*< .05 indicates statistical significance.

Bold values are statistically significant (*p*< 0.05).

### 3.3 Associations of *MMP8*/*MMP10* polymorphisms with risk factors for IS

Along with a stratified analysis based on age, gender, smoking, and alcohol intake, we further evaluated the association of *MMP8*/*MMP10* polymorphisms with risk factors for IS. The analysis stratified by age showed that rs17860949 was associated with a decreased susceptibility to IS in people aged >55 years in the allele (OR = 0.472, 95% CI = 0.311–0.715, *p* < .001), codominant (OR = 0.495, 95% CI = 0.301–0.814, *p* = .006), dominant (OR = 0.480, 95% CI = 0.293–0.787, *p* = .004), and log‐additive models (OR = 0.477, 95% CI = 0.294–0.774, *p* = .003) (Table 5). After stratification by gender (Table 6), rs17860949 was related to a decreased susceptibility to IS in males (allele: OR = 0.632, 95% CI = 0.441–0.905, *p* = .012; codominant: OR = 0.647, 95% CI = 0.438–0.955, *p* = .028; dominant: OR = 0.636, 95% CI = 0.433–0.935, *p* = .021; and log‐additive: OR = 0.642, 95% CI = 0.443–0.929, *p* = .019). As summarized in Table 7, rs17860949 was related to a lower risk of IS in nonsmokers (allele: OR = 0.610, 95% CI = 0.405–0.919, *p* = .017 and log‐additive: OR = 0.636, 95% CI = 0.415–0.975, *p* = .038). Furthermore, rs17860949 had a risk‐decreasing impact on IS in nondrinkers (allele: OR = 0.559, 95% CI = 0.369–0.847, *p* = .006; codominant: OR = 0.602, 95% CI = 0.384–0.946, *p* = .028; dominant: OR = 0.579, 95 %CI = 0.370–0.905, *p* = .016; and log‐additive: OR = 0.569, 95% CI = 0.369–0.880, *p* = .011).

**TABLE 5:** Associations of MMP8/MMP10 polymorphisms with ischemic stroke risk stratified by age

SNP ID	Model	Genotype	Case	Control	OR (95% CI)	*p*	Case	Control	OR (95% CI)	*p*
			>55 years				≤55 years			
rs1940475	Allele	*C*	500	374	1		373	507	1	
		*T*	292	242	0.903 (0.727–1.121)	.354	229	275	1.132 (0.908–1.411)	.271
	Codominant	*TC*	176	144	0.872 (0.601–1.266)	.473	135	179	1.034 (0.742–1.439)	.843
		*TT*	58	49	0.933 (0.557–1.563)	.792	47	48	1.372 (0.852–2.209)	.194
		*CC*	162	115	1		119	164	1	
	Dominant	*TC*‐*TT*	234	193	0.887 (0.625–1.260)	.503	182	227	1.104 (0.808–1.508)	.536
	Recessive	*CC*‐*TC*	338	259	1		254	343	1	
		*TT*	58	49	1.001 (0.625–1.614)	.985	47	48	1.348 (0.866–2.098)	.187
	Log‐additive	–	–	–	0.945 (0.740–1.206)	.647	–	–	1.135 (0.907–1.418)	.268
rs3765620	Allele	*A*	507	383	1		384	514	1	
		*G*	287	235	0.923 (0.742–1.147)	.468	220	268	1.099 (0.880–1.372)	.405
	Codominant	*AG*	171	143	0.868 (0.600–1.258)	.456	128	172	1.002 (0.721–1.393)	.990
		*GG*	58	46	0.998 (0.592–1.681)	.993	46	48	1.297 (0.807–2.085)	.283
		*AA*	168	120	1		128	171	1	
	Dominant	*AG*‐*GG*	229	189	0.899 (0.635–1.273)	.548	174	220	1.066 (0.782–1.451)	.687
	Recessive	*AA*‐*AG*	339	263	1		256	343	1	
		*GG*	58	46	1.074 (0.662–1.743)	.772	46	48	1.296 (0.831–2.021)	.253
	Log‐additive	–	–	–	0.966 (0.757–1.234)	.783	–	–	1.101 (0.883–1.374)	.393
rs17860949	Allele	*G*	755	557	1		563	726	1	
		*A*	39	61	0.472 (0.311–0.715)	**< .001**	37	56	0.852 (0.555–1.309)	.465
	Codominant	*AG*	39	55	0.495 (0.301–0.814)	**.006**	35	54	0.800 (0.504–1.270)	.344
		*AA*	0	3	/	/	1	1	1.143 (0.071–18.520)	.925
		*GG*	358	251	1		264	336	1	
	Dominant	*AG*‐*AA*	39	58	0.480 (0.293–0.787)	**.004**	36	55	0.807 (0.511–1.274)	.357
	Recessive	*GG*‐*AG*	397	306	1		299	390	1	
		*AA*	0	3	/	/	1	1	1.178 (0.073–19.060)	.908
	Log‐additive	–	–	–	0.477 (0.294–0.774)	**.003**	–	–	0.822 (0.528–1.278)	.383

OR, odds ratio, 95 % CI; 95% confidence intervals.

The *p* values were calculated by logistic regression with adjustments for gender, smoking, and drinking.

The *p* < .05 indicates statistical significance.

Bold values are statistically significant (*p*< 0.05).

**TABLE 6:** Associations of MMP8/MMP10 polymorphisms with ischemic stroke risk stratified by gender

SNP ID	Model	Genotype	Case	Control	OR (95% CI)	*p*	Case	Control	OR (95% CI)	*p*
			Male				Female			
rs1940475	Allele	*C*	574	567	1		299	314	1	
		*T*	340	345	0.974 (0.806–1.177)	.781	181	172	1.105 (0.850–1.436)	.455
	Codominant	*TC*	206	211	0.966 (0.725–1.288)	.814	105	112	1.068 (0.714–1.596)	.750
		*TT*	67	67	1.001 (0.668–1.501)	.996	38	30	1.333 (0.752–2.366)	.325
		*CC*	184	178	1		97	101	1	
	Dominant	*TC*‐*TT*	273	278	0.975 (0.743–1.278)	.852	143	142	1.127 (0.772–1.644)	.537
	Recessive	*CC*‐*TC*	390	389	1		202	213	1	
		*TT*	67	67	1.020 (0.701–1.483)	.919	38	30	1.289 (0.756–2.198)	.352
	Log‐additive	–	–	–	0.992 (0.820–1.201)	.936	–	–	1.132 (0.866–1.481)	.364
rs3765620	Allele	*A*	590	580	1		301	317	1	
		*G*	328	334	0.965 (0.798–1.168)	.717	179	169	1.115 (0.858–1.451)	.415
	Codominant	*AG*	196	202	0.961 (0.722–1.280)	.787	103	113	0.984 (0.660–1.467)	.938
		*GG*	66	66	0.975 (0.651–1.461)	.902	38	28	1.356 (0.759–2.422)	.303
		*AA*	197	189	1		99	102	1	
	Dominant	*AG*‐*GG*	262	268	0.965 (0.738–1.261)	.793	141	141	1.062 (0.729–1.545)	.755
	Recessive	*AA*‐*AG*	393	391	1		202	215	1	
		*GG*	66	66	0.995 (0.682–1.450)	.978	38	28	1.367 (0.794–2.353)	.259
	Log‐additive	–	–	–	0.981 (0.812–1.185)	.842	–	–	1.114 (0.851–1.457)	.432
rs17860949	Allele	*G*	863	833	1		455	450	1	
		*A*	53	81	0.632 (0.441–0.905)	**.012**	23	36	0.632 (0.369–1.083)	.093
	Codominant	*AG*	51	75	0.647 (0.438–0.955)	**.028**	23	34	0.710 (0.395–1.276)	.252
		*AA*	1	3	0.360 (0.037–3.512)	.380	0	1	/	/
		*GG*	406	379	1		216	208	1	
	Dominant	*AG*‐*AA*	52	78	0.636 (0.433–0.935)	**.021**	23	35	0.689 (0.385–1.235)	.211
	Recessive	*GG*‐*AG*	457	454	1		239	242	1	
		*AA*	1	3	0.383 (0.039–3.727)	.408	0	1	/	/
	Log‐additive	–	–	–	0.642 (0.443–0.929)	**.019**	–	–	0.676 (0.382–1.195)	.178

OR, odds ratio, 95 % CI; 95% confidence intervals.

The *p* values were calculated by logistic regression with adjustments for age, smoking, and drinking.

The *p* < .05 indicates statistical significance.

Bold values are statistically significant (*p*< 0.05).

**TABLE 7:** Associations of MMP8/MMP10 polymorphisms with ischemic stroke stratified by smoking and alcohol intake

SNP ID	Model	Genotype	Smoking		Nonsmoking		Alcohol intake		No alcohol intake	
			OR (95% CI)	*p*	OR (95% CI)	*p*	OR (95% CI)	*p*	OR (95% CI)	*p*
rs1940475	Allele	*C*	1		1		1		1	
		*T*	1.054 (0.846–1.313)	.642	0.984 (0.794–1.219)	.882	0.983 (0.789–1.224)	.875	1.051 (0.848–1.302)	.651
	Codominant	*TC*	1.015 (0.723–1.426)	.932	1.031 (0.747–1.422)	.853	0.926 (0.661–1.297)	.653	1.146 (0.827–1.589)	.412
		*TT*	1.262 (0.791–2.015)	.330	1.041 (0.652–1.660)	.867	1.225 (0.763–1.965)	.401	1.103 (0.694–1.754)	.679
		*CC*	1		1		1		1	
	Dominant	*TC*‐*TT*	1.073 (0.780–1.476)	.666	1.033 (0.763–1.400)	.833	0.992 (0.722–1.362)	.959	1.136 (0.835–1.545)	.418
	Recessive	*CC*‐*TC*	1		1		1		1	
		*TT*	1.252 (0.814–1.925)	.306	1.024 (0.663–1.583)	.914	1.276 (0.824–1.977)	.274	1.025 (0.667–1.576)	.909
	Log‐additive	–	1.099 (0.880–1.373)	.407	1.023 (0.823–1.272)	.838	1.061 (0.849–1.327)	.601	1.073 (0.863–1.335)	.527
rs3765620	Allele	*A*	1		1		1		1	
		*G*	1.083 (0.868–1.351)	.482	0.955 (0.770–1.184)	.672	1.036 (0.831–1.292)	.753	0.996 (0.802–1.236)	.970
	Codominant	*AG*	1.093 (0.780–1.532)	.607	0.912 (0.662–1.257)	.573	1.019 (0.729–1.425)	.911	0.978 (0.707–1.353)	.893
		*GG*	1.291 (0.809–2.060)	.285	1.008 (0.631–1.611)	.974	1.254 (0.779–2.020)	.352	1.068 (0.674–1.694)	.779
		*AA*	1		1		1		1	
	Dominant	*AG*‐*GG*	1.141 (0.832–1.564)	.414	0.933 (0.690–1.262)	.654	1.072 (0.782–1.469)	.667	1.000 (0.738–1.355)	.998
	Recessive	*AA*‐*AG*	1		1		1		1	
		*GG*	1.233 (0.799–1.904)	.344	1.056 (0.681–1.640)	.807	1.242 (0.798–1.932)	.338	1.080 (0.701–1.663)	.727
	Log‐additive	–	1.126 (0.903–1.404)	.292	0.978 (0.787–1.215)	.840	1.094 (0.875–1.368)	.430	1.019 (0.822–1.264)	.863
rs17860949	Allele	*G*	1		1		1		1	
		*A*	0.658 (0.426–1.018)	.059	0.610 (0.405–0.919)	**.017**	0.723 (0.470–1.112)	.138	0.559 (0.369–0.847)	**.006**
	Codominant	*AG*	0.670 (0.417–1.077)	.098	0.677 (0.434–1.055)	.085	0.740 (0.463–1.183)	.209	0.602 (0.384–0.946)	**.028**
		*AA*	1.485 (0.092–24.000)	.781	/	/	1.519 (0.094–24.58)	.769	/	/
		*GG*	1		1		1		1	
	Dominant	*AG*‐*AA*	0.683 (0.427–1.091)	.111	0.650 (0.419–1.009)	.055	0.753 (0.473–1.197)	.230	0.579 (0.370–0.905)	**.016**
	Recessive	*GG*‐*AG*	1		1		1		1	
		*AA*	1.572 (0.097–25.400)	.750	/	/	1.584 (0.098–25.62)	.746	/	/
	Log‐additive	–	0.707 (0.449–1.115)	.136	0.636 (0.415–0.975)	**.038**	0.775 (0.494–1.216)	.267	0.569 (0.369–0.880)	**.011**

OR, odds ratio, 95 % CI; 95% confidence intervals.

The *p* values were calculated by logistic regression with adjustments for age and gender.

The *p* < .05 indicates statistical significance.

Bold values are statistically significant (*p*< 0.05).

### 3.4 FPRP results

The FPRP test was performed to verify positive results, and the threshold was set as 0.2. As shown in Table S1, at the prior probability of 0.25, all the positive findings of the correlation between *MMP10* rs17860949 and IS risk remained noteworthy (FPRP < 0.2).

### 3.5 The impacts of SNPs on clinical indicators of IS patients

As demonstrated in Table 8, GG genotype (1.760 ± 1.130 mmol/L) and AG genotype (1.502 ± 0.694 mmol/L) in rs3765620 were related to a reduced triglycerides concentration in IS patients compared with AA genotype (1.973 ± 1.363 mmol/L) (*p* = .018).

**TABLE 8:** Comparisons between clinical indicators and SNP genotypes in ischemic stroke patients

SNP	Total cholesterol (mmol/L)	Triglycerides (mmol/L)	HDL‐C (mmol/L)	LDL‐C (mmol/L)
rs1940475				
*TT*	3.940 ± 0.893	1.812 ± 1.141	1.068 ± 0.179	1.825 ± 0.662
*CT*	3.948 ± 1.011	1.602 ± 0.805	1.098 ± 0.214	2.021 ± 0.760
*CC*	3.974 ± 1.050	1.909 ± 1.386	1.111 ± 0.290	1.920 ± 0.581
*p*	.981	.186	.745	.368
rs3765620				
*AA*	3.971 ± 1.056	1.973 ± 1.363	1.101 ± 0.284	1.952 ± 0.611
*AG*	3.945 ± 0.994	1.502 ± 0.694	1.107 ± 0.212	2.004 ± 0.755
*GG*	3.970 ± 0.855	1.760 ± 1.130	1.072 ± 0.176	1.885 ± 0.693
*p*	0.984	**0.018**	0.807	0.715
rs17860949				
*AG*	4.056 ± 1.162	1.840 ± 1.353	1.060 ± 0.233	2.088 ± 0.580
*GG*	3.952 ± 0.987	1.729 ± 1.084	1.105 ± 0.245	1.953 ± 0.696
*p*	.656	.666	.426	.393

The *p* values were calculated by Kruskal–Wallis *H* test.

The *p* < .05 indicates statistical significance.

Bold values are statistically significant (*p*< 0.05).

## 4 DISCUSSION

Stroke is one of the major causes of mortality, long‐term physical and cognitive impairment in China (Tu et al., 2019). Stroke prevention and treatment has been listed as an important campaign of the Healthy China Initiative. The China Stroke Prevention Project Committee (CSPPC) aims to reduce the incidence and mortality of stroke by establishing stroke map, organizing health education and professional training, screening high‐risk populations, and conducting follow‐up (Chao et al., 2021). Notably, stroke is a polygenic and multifactorial disease. Genetic factors, like gene polymorphisms, play an important role in occurrence of stroke (Syahrul et al., 2018; Worrall et al., 2007), and may also exert a protective effect against stroke. Our study investigated the association of *MMP8*/*MMP10* polymorphisms with the susceptibility to IS in the Shaanxi Han population, suggesting that the rs17860949 polymorphism in *MMP10* was associated with a decreased susceptibility to IS.

The rs17860949 polymorphism is located on the second exon of *MMP10*. In our research, rs17860949 could reduce the risk of IS. However, Zhu has observed that *MMP10* rs17435959 and rs17293607 are independent of susceptibility to atherothrombotic cerebral infarction in Zhejiang Han population (Zhu et al., 2013). Wu et al.’s (2021)’s study has indicated that *MMP10* rs17435959 is related to the stability and formation of carotid atherosclerosis plaque. Besides, we observed that rs17860949 in *MMP10* could have an influence on IS risk in people aged > 55 years, suggesting that there were age differences in the impact of rs17860949 on the risk of IS. Cai et al. (2020) have reported that rs4646 impacts on stroke susceptibility in people aged > 64 years. Besides, Wu et al. (2020) have showed that genetic polymorphisms are significantly associated with stroke risk in patients aged > 65 years. However, Yang et al. (2020) has found that genetic variants influence stroke risk in patients aged ≤ 64 years. Wang et al. (2019) has reported that gene polymorphism is related to stroke risk in people aged < 60 years. Age is a risk factor for stroke, and the incidence of stroke increases with age, doubling every decade after age 55 (Roger et al., 2012). Taken together, these findings indicate that genetic susceptibility to stroke is influenced by age and highlight the importance of heterogeneity in studies of the association between genetic factors and stroke.

In addition, we observed that the rs17860949 polymorphism was associated with the risk of IS in men but not in women. Similar to our results, Gu et al. (2018) and Titov et al. (2016) have reported that polymorphisms are related to stroke susceptibility in men. On the contrary, Gu et al. (2018) and Xu et al. (2017) have observed that gene polymorphism can have an impact on IS risk in women. These results suggest that the impact of genetic polymorphisms on IS may be dependent on gender. In other words, gender is a nonmodifiable risk factor for stroke. The prevalence of stroke in men is higher than that in women, but the incidence and mortality of stroke in older men are lower than those in older women, suggesting that there are congenital differences in the occurrence of stroke between women and men (Petrea et al., 2009; Wang et al., 2014). In addition, studies have found that gender differences in stroke may be related to factors such as age, sex hormones, genetics, and lifestyle (Appelros & Åsberg, 2020; Samai & Martin‐Schild, 2015).

We further investigated the relationship of polymorphisms with IS risk after stratified by modifiable risk factors, such as smoking and alcohol consumption. Our analysis showed that rs17860949 has a protective role in IS in nonsmokers and nondrinkers. O¨zcelik has revealed that variants could increase the susceptibility to IS in smokers (Türkanoğlu Özçelik et al., 2017; Türkanoğlu Özçelik et al., 2018). Lin and Kamdee have reported that gene polymorphisms significantly increase the susceptibility to IS in drinkers (Kamdee et al., 2021; Lin et al., 2021). These results indicated that the impact of gene polymorphisms on IS risk may be linked to smoking and alcohol consumption.

Triglyceride is an important component of blood lipids and triglyceride is a component of dyslipidemia (Dron & Hegele, 2017).  Triglyceride levels are risk factors for IS (Iso et al., 2014; Tanne et al., 2001). Dziedzic et al. (2004) have found that lower triglyceride concentrations are related to an increased severity of stroke. We discovered that GG genotype and AG genotype in *MMP8* rs3765620 were related to a reduced triglyceride level, which means this mutation may accelerate the occurrence of IS.

Our study has some limitations. First, the association between genetic polymorphism and *MMP8/MMP10* gene expression is not evaluated in the present study, and it will be investigated in next studies. Second, risk factors for IS, such as hypertension, diabetes, and hyperlipidemia, were not obtained, which limited our ability to assess the effect of interactions between gene and environment on IS risk. Third, we did not explore the molecular mechanism of *MMP8/MMP10* polymorphisms in IS.

## 5 CONCLUSION

*MMP10* rs17860949 can impact the risk of IS in the Shaanxi Han population. More interestingly, GG genotype and AG genotype in *MMP8* rs3765620 are related to a reduced triglyceride level in patients with IS.

## FUNDING

This work was supported by Natural Science Foundation of China (No. 82104155‐Wenzhen Shi), Key Research and Development Program of Shaanxi (2020ZDLSF04‐03‐Ye Tian and 2021SF‐096‐Wenzhen Shi), and Xi'an Science and Technology Planning Project (21YXYY0038‐Wenzhen Shi and 21YXYJ0004‐Mingze Chang).

## CONFLICT OF INTEREST

All authors declared that they have no conflict of interests.

### ETHICAL STATEMENT

All procedures involving human participants performed in this study were in accordance with the ethical standards of Xi'an No. 3 Hospital, the Affiliated Hospital of Northwest University and the Helsinki's Declaration. Informed consent was obtained from all individual participants.

### PEER REVIEW

The peer review history for this article is available at https://publons.com/publon/10.1002/brb3.2797

## Supporting information

Table S1 False‐positive report probability analysis for the positive findings between *MMP8*/*MMP10* polymorphisms and ischemic stroke risk

## Data Availability

Participant informed consent statements did not seek consent for data to be made publicly available; however, data will be made available to individual researchers upon reasonable request.
