# Supplementary material for: The roles of MMP8/MMP10 polymorphisms in ischemic stroke susceptibility
Source: Brain Behav. 2022 Oct 25;12(12):e2797. doi: 10.1002/brb3.2797 (PMC9759140; doi:10.1002/brb3.2797)
Supplement: Supplementary file 1 — Table S1 False‐positive report probability analysis for the positive findings between MMP8/MMP10 polymorphisms and ischemic stroke risk [file BRB3-12-e2797-s001.docx]

Table S1 False-positive report probability analysis for the positive findings between *MMP8*/*MMP10* polymorphisms and ischemic stroke risk

| Genotype and Variables | OR (95 % CI) | *p* Value ^a^ | Statistical Power ^b^ | Prior Probability | | | |  |
| --- | --- | --- | --- | --- | --- | --- | --- | --- |
|  |  |  |  | 0.25 | 0.1 | 0.01 | 0.001 | 0.0001 |
| rs17860949 G > *A* |  |  |  |  |  |  |  |  |
| *A* Vs *G* | 0.632 (0.469-0.853) | 0.002 | 0.937 | 0.009 ^c^ | 0.025 ^c^ | 0.222 | 0.743 | 0.967 |
| *AG* Vs *GG* | 0.641 (0.467-0.879) | 0.006 | 0.938 | 0.018 ^c^ | 0.052 ^c^ | 0.378 | 0.860 | 0.984 |
| *AG*-*AA* Vs *GG* | 0.627 (0.458-0.858) | 0.004 | 0.921 | 0.011 ^c^ | 0.033 ^c^ | 0.275 | 0.793 | 0.975 |
| **Age> 55 years** |  |  |  |  |  |  |  |  |
| rs17860949 G > *A* |  |  |  |  |  |  |  |  |
| *A* Vs *G* | 0.472 (0.311-0.715) | < 0.001 | 0.393 | 0.003 ^c^ | 0.009 ^c^ | 0.091 ^c^ | 0.501 | 0.910 |
| *AG* Vs *GG* | 0.495 (0.301-0.814) | 0.006 | 0.484 | 0.033 ^c^ | 0.094 ^c^ | 0.533 | 0.920 | 0.991 |
| *AG*-*AA* Vs *GG* | 0.480 (0.293-0.787) | 0.004 | 0.436 | 0.024 ^c^ | 0.070 ^c^ | 0.451 | 0.892 | 0.988 |
| **male** |  |  |  |  |  |  |  |  |
| rs17860949 G > *A* |  |  |  |  |  |  |  |  |
| *A* Vs *G* | 0.632 (0.441-0.905) | 0.012 | 0.900 | 0.039 ^c^ | 0.109 ^c^ | 0.574 | 0.932 | 0.993 |
| *AG* Vs *GG* | 0.647 (0.438-0.955) | 0.028 | 0.903 | 0.086 ^c^ | 0.221 | 0.757 | 0.969 | 0.997 |
| *AG*-*AA* Vs *GG* | 0.636 (0.433-0.935) | 0.021 | 0.889 | 0.067 ^c^ | 0.178 ^c^ | 0.704 | 0.960 | 0.996 |
| **Non-smoking** |  |  |  |  |  |  |  |  |
| rs17860949 G > *A* |  |  |  |  |  |  |  |  |
| *A* Vs *G* | 0.610 (0.405-0.919) | 0.017 | 0.819 | 0.079 ^c^ | 0.204 | 0.738 | 0.966 | 0.996 |
| **No alcohol intake** |  |  |  |  |  |  |  |  |
| rs17860949 G > *A* |  |  |  |  |  |  |  |  |
| *A* Vs *G* | 0.559 (0.369-0.847) | 0.006 | 0.670 | 0.089 ^c^ | 0.227 | 0.764 | 0.970 | 0.997 |
| *AG* Vs *GG* | 0.602 (0.384-0.946) | 0.028 | 0.857 | 0.012 ^c^ | 0.036 ^c^ | 0.292 | 0.807 | 0.977 |
| *AG*-*AA* Vs *GG* | 0.579 (0.370-0.905) | 0.016 | 0.721 | 0.1082 ^c^ | 0.267 | 0.800 | 0.976 | 0.998 |

*p* value ^a^ was calculated by unconditional logistic regression analysis with adjustment for age and gender.

Statistical power ^b^ was calculated using the number of observations in the subgroup and the OR and *p* values in this table.

^c^ The level of false-positive report probability threshold was set at 0.2 and noteworthy findings are presented.
